# An fMRI study of visual geometric shapes processing

**DOI:** 10.3389/fnins.2023.1087488

**Published:** 2023-03-16

**Authors:** Liuqing Wei, Xueying Li, Lina Huang, Yuansheng Liu, Luming Hu, Wenbin Shen, Qingguo Ding, Pei Liang

**Affiliations:** ^1^Department of Psychology, Faculty of Education, Hubei University, Wuhan, China; ^2^Brain and Cognition Research Center, Faculty of Education, Hubei University, Wuhan, China; ^3^Imaging Department, Changshu No. 2 People’s Hospital, The Clinical Medical College Affiliated to Xuzhou Medical University, Changshu, China; ^4^Department of Psychology, School of Arts and Sciences, Beijing Normal University, Zhuhai, China

**Keywords:** vision, circular and angular shapes, emotion, fMRI, cross-modal correspondence

## Abstract

Cross-modal correspondence has been consistently evidenced between shapes and other sensory attributes. Especially, the curvature of shapes may arouse the affective account, which may contribute to understanding the mechanism of cross-modal integration. Hence, the current study used the functional magnetic resonance imaging (fMRI) technique to examine brain activity’s specificity when people view circular and angular shapes. The circular shapes consisted of a circle and an ellipse, while the angular shapes consisted of a triangle and a star. Results show that the brain areas activated by circular shapes mainly involved the sub-occipital lobe, fusiform gyrus, sub and middle occipital gyrus, and cerebellar VI. The brain areas activated by angular shapes mainly involve the cuneus, middle occipital gyrus, lingual gyrus, and calcarine gyrus. The brain activation patterns of circular shapes did not differ significantly from those of angular shapes. Such a null finding was unexpected when previous cross-modal correspondence of shape curvature was considered. The different brain regions detected by circular and angular shapes and the potential explanations were discussed in the paper.

## Introduction

Plenty of literature has provided evidence that shapes’ curvilinearity (roundedness and angularity) may have consistent cross-modal correspondences with other sensory attributes (Spence, 2011; Spence and Ngo, 2012; Ghoshal et al., 2016; Lee and Spence, 2022). For instance, people associate curved shapes with a sweet taste, quiet or calm sound, vanilla smell, green color, smooth texture, relieved emotion, female gender, and wide-vowel names; while they associate angular shapes with sour taste, loud or dynamic sound, spicy or citrus smell, red color, rough texture, excited or surprise emotion, male gender, and narrow-vowel names (Blazhenkova and Kumar, 2017). Different hypotheses have been proposed for the cross-modal correspondence effect (Lee and Spence, 2022). One is the statistical account, which means the internalization of the multisensory statistics of the environment. The other is the affective account, and cross-modal correspondences are mediated by emotion (Wang et al., 2016). Thus, an intriguing question arises: how does the brain process geometric shapes, particularly circular and angular shapes?

People generally prefer circular stimuli over angular ones (Wang and Zhang, 2016; Cotter et al., 2017). The geometric stimuli may convey different emotions. For example, diagonal and angular shapes tend to be associated with threat, while rounded features and curved lines are associated with pleasure and happiness (Bar and Neta, 2007, 2016; Larson et al., 2012). At the behavioral level, Bar and Neta (2016) found that people disliked sharp-angled neutral objects more than curved neutral objects. They proposed that people may perceive the sharp contour as threatening, thus influencing their attitudes toward sharp-angled objects to be negative (Bar and Neta, 2016). Bar and Neta (2007) found that compared to objects with curving contours, neutral objects (abstract figures, everyday objects) containing sharp contours elicited greater activation of the amygdala, a brain structure that involves in fear processing and is proportional to arousal in genera. Larson et al. (2016) also validated the greater activation of the amygdala by the presentation of downward-pointing V-shapes, compared to the identical V-shape pointing upward. Moreover, recent electrophysiological evidence has shown that ellipse and triangle shapes were found to arouse similar ERP responses (N1, N2, P1, and P2 recorded from parietal lobes) to line-drawn happy and angry faces (Li et al., 2018). These findings suggest that simple shapes can induce threat and negative affection at the low-level perceptual stage.

Recently, the brain areas involved in geometric shape processing have been proposed in a distributed network (Freud et al., 2017; Freud and Behrmann, 2020) or *via* a skeletal structure located in V3 and lateral occipital cortex for perceptual organization and object recognition (Ayzenberg et al., 2019a,b; Ayzenberg and Lourenco, 2019). Bracci and de Beeck (2016) found that shape and category information interacts throughout the ventral and dorsal visual pathways for successful object recognition. Ayzenberg et al. (2022) used models that approximate early-, mid- and high-level visual processing. Indeed, V3 has been consistently implicated in creating shape percepts (Montaser-Kouhsari et al., 2007; Caplovitz et al., 2008; McMains and Kastner, 2010). Perceptual organization is accomplished by border ownership cells in V2 and the subsequent visual region V3, to specify the contours of a figure (Zhou et al., 2000; von der Heydt, 2015). Temporal dynamics analysis has shown that low-level properties like contrast and spatial frequencies, medial-axis like shape and category have an independent process at the early time window (100–150 ms) (Papale et al., 2019). Hence, the object shape is encoded along different dimensions and each representing orthogonal features (Papale et al., 2020).

Based on the above research results, geometric information is not only processed according to the statistical account throughout the visual cortex. The affective account of the geometric shapes is also involved in different stages of visual pathways. In particular, shapes’ curvilinearity (roundedness and angularity) can lead to different preferences and affective effects with cross-modal experiments (Velasco et al., 2015). What brain areas are involved in the processing of circular and angular shapes? Is shape curvature processed differently by different regions? This study aimed to answer these questions. Using the fMRI technique, the current study adopted a simple geometric shape viewing task and explored the brain activation of simple circular and angular shapes processing. It was expected to find different activation of brain areas for circular and angular shapes. If yes, which brain regions are involved and the possible explanation will be discussed later.

## Materials and methods

### Participants

In our experiment, a total of 12 subjects volunteered to participate in the experiment, including three males and nine females. The average age of the participants is 21.75 ± 0.62 (from 21 to 23) years old. All participants had normal or corrected to normal vision and reported no history of neurological or psychiatric disorder. All participants were well informed about the study’s procedures and provided informed written consent. The study was approved by the Ethics Committee of the No. 2 People’s Hospital of Changshu (license number 2018–68). All participants received payments for their time.

### Stimuli

**FIGURE 1:**
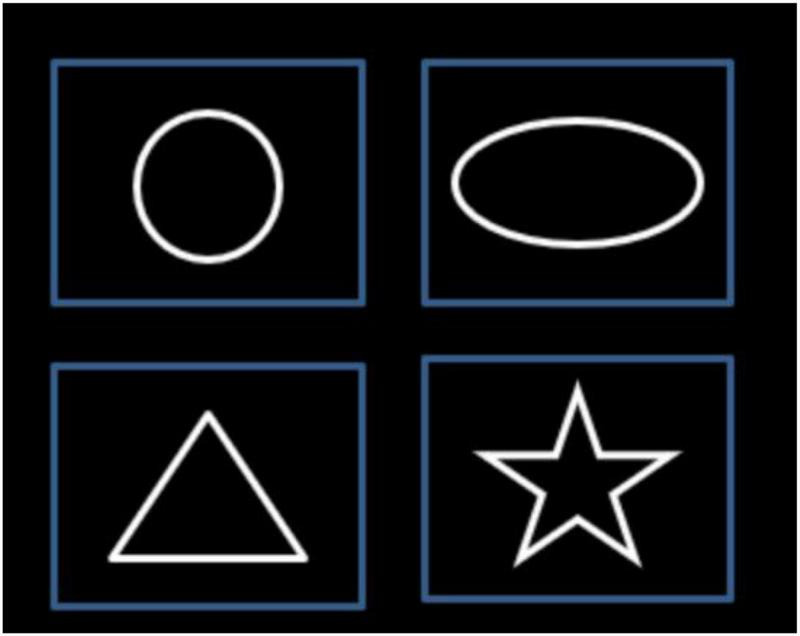
Four shapes, circle and ellipse as circular type, and triangle and star as angular type. Each of the shapes was displayed randomly as visual stimuli in the experiment.

The basic geometric shapes with different curvatures were applied. Circular shapes included a circle and an ellipse. Angular shapes contain triangles and stars. The two types of shapes are comparable in contrast, color, and size when presented on display (Figure 1).

### Tasks

The experiment contained four blocks, and each block included six trials. In each trial, participants watched one geometric shape for 6 s, then rested for 14 s. Each block repeated, displaying the same shape six times and lasted 120 s. The whole experiment lasted around 8 min. The order of the blocks was balanced among the participants.

### MRI data acquisition

Structural and functional MRI data were collected using the GE Discovery MR750W 3.0T scanning system. The scanner is with an 8-channel head coil. During the scanning process, the participants quietly lay on the back of the magnetic resonance examination bed, fixing the head inside the head coil with foam padding, and wearing earplugs to reduce machine noise. The participants watched the visual stimuli through a reflector mirror mounted on the head coil. Through the mirror, the visual stimuli were reflected from the projector screen, placed outside the gate of the MRI. For structural imaging, high-resolution T1 weighted scans were acquired. The 3D T1 BRAVO_SPM volume sequence was applied with TR = 8.5 ms, TE = 3.2 ms, flip angle = 12°, FOV = 24 cm, 256 × 256 matrix size, and 1 mm slice thickness. The MR sequence for T2-weighted functional imaging was acquired using TR = 2000 ms, TE = 30 ms, flip angle = 90°, scan resolution of 64 × 64, 33 slices, intervals of 2 mm with slice thickness = 3.6 mm, FOV = 24 cm, voxel size = 3.75 × 3.75 × 3.75 mm^3^.

### fMRI data analysis

#### Shape fMRI data analysis

The preprocessing and statistical analysis of NMR data mainly used the SPM12 toolbox (Wellcome Department of Imaging Neuroscience, University College London, UK)^1^ and DPAIBI toolbox (a toolbox for Data Processing and Analysis for Brain Imaging, China)^2^ based on Matlab 2012b (Mathworks, MA)^3^ platform.

The preprocessing of functional image data includes head movement correction, tissue segmentation, spatial registration and spatial Standardization (mapped to MNI (Montreal Neurological Institute)) standard space established by Montreal Institute of Neurology [bounding box: – 126 – 72; 90 108; – 90 90 90], with a resolution of 3 × 3 × 3 mm^3^, and spatial smoothing (using a three-dimensional Gaussian kernel with a half height and width of 6 mm). Among them, the sequences in which the translation amount of the head movement range exceeds 2 mm or the rotation amount exceeds 2° are eliminated (around 6% of sequences are eliminated).

After the pretreatment, in the first-order analysis, the design parameters of the three conditions are combined with Hemodynamic Response Function (HRF) to construct the parameter model of a unified general linear model (GLM), and the head movement parameters are included in the model as covariates. At the first-order individual level, four groups of comparative analysis were carried out, namely geometric shapes (circular + angular) > resting condition, circular condition > resting condition, angular condition > resting condition, and circular condition > angular condition.

At the second-order group level, the four groups for each participant were grouped and combined by DPABI software, and then the group single sample *t*-test was performed. In the group single sample *t*-test, the non-parametric permutation test method is used for treatment, the number of permutations is 5000, and the cluster-forming threshold is set to *P* < 0.001. Finally, voxels with *P* < 0.05 are presented. All the above comparisons were performed on a custom gray matter removal template. The whole brain activation map was rendered by BrainNet view software.

## Results

We compared circular shapes vs angular shapes first. However, no significant brain activation was detected (we presented the details of the null results in the Supplementary material). The results would be insignificant if the voxels of the detected brain regions were below 10. Hence, we looked at the common brain regions stimulated by geometric shapes and the regions by circular and angular shapes separately.

### Analysis of common brain regions stimulated by geometric shapes

**FIGURE 2:**
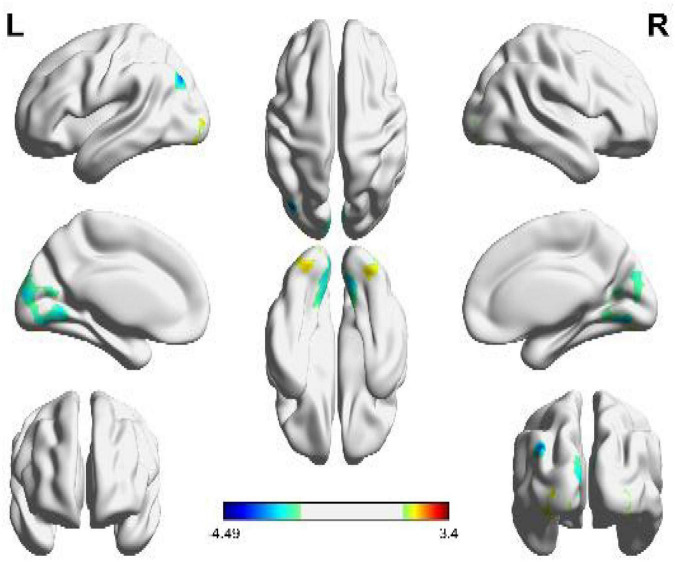
Brain activation regions of geometric shapes (circular and angular shapes). The color bar indicates the size of the activation intensity. The values indicate positive or negative activation.

Through the whole brain fMRI analysis, we analyzed the activated brain regions by viewing geometric shapes (circular shapes and angular shapes). We obtained the three-dimensional brain map, as shown in Figure 2. The details of the active voxel group in the figure are shown in Table 1.

**TABLE 1:** Clusters of brain activation areas for geometric shapes.

Labels	Brain side	Voxels	*t*	MNI		
				***x***	***y***	***z***
Inferior occipital gyrus	L	190	3.395	−24	−90	−3
Middle occipital gyrus	L	102	−4.487	−39	−78	36
Fusiform gyrus	R	152	2.704	30	−84	−6
Cerebellum (VI)	R	1151	−2.962	12	−72	−9
Cerebellum (VI)	L	1151	−2.669	−15	−72	−15
Cerebellum (VII)	L	11	−1.995	−21	−75	−42
Cuneus	L	1151	−2.822	3	−87	27
Precuneus	L	17	−2.675	−3	−54	69
Precuneus	L	17	−1.972	0	−72	51
Caudate nucleus	R	11	−2.138	21	−9	27

Tables 1–3, L, left brain; R, right brain. *t*-value shows the mean difference of activation.

As can be seen from Figure 2, the brain activation functional areas of the geometric shapes are mainly located in the cuneus, cerebellum VI, fusiform gyrus, middle occipital gyrus and suboccipital gyrus, followed by pre-cuneus lobe and cerebellum VII.

According to the details of the activated voxel group in Table 1, it can be seen that the maximum range includes 1151 voxels, including the cuneus and cerebellar VI. This is because the cuneus is related to basic visual processing; Cerebellar VI is related to motor planning and coordination. Secondly, the fusiform, suboccipital, and middle occipital gyrus were activated. This is because the function of the fusiform gyrus is related to object and face recognition (Keshav and Hof, 2013). The middle occipital gyrus is the brain region of early visual processing, mainly responsible for extracting shapes and external visual features of words and objects (Beharelle and Small, 2016).

### Analysis of independent brain regions stimulated by different shapes

**FIGURE 3:**
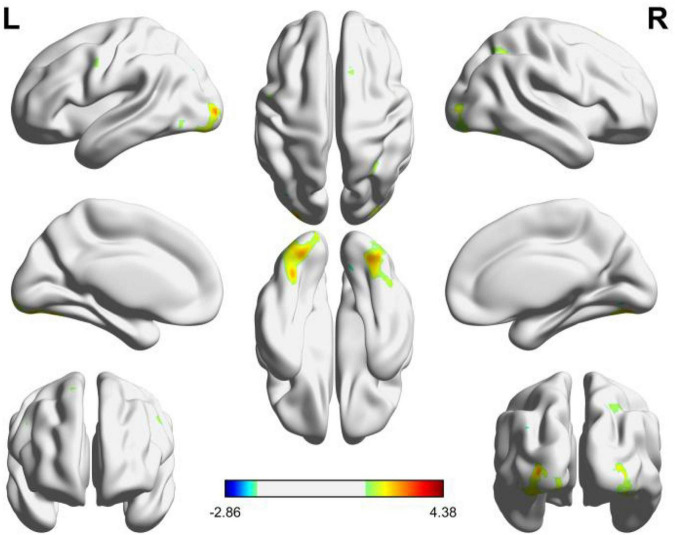
Brain functional areas activated by circular shapes. The color bar indicates the size of the activation intensity. The values indicate positive or negative activation.

In order to further examine the brain regions excited by circular and angular shapes, the brain activation maps of the two shapes were obtained, respectively. As shown in Figure 3, it is the activation of circular shapes in the brain region. The details of activated voxel groups are shown in Table 2.

**TABLE 2:** Clusters of brain areas activated by circular shapes.

Labels	Brain side	Voxels	*t*	MNI		
				***x***	***y***	***z***
Inferior occipital	L	454	4.381	−21	−93	0
Inferior occipital gyrus	R	346	3.174	30	−93	0
Middle occipital gyrus	L	17	−2.864	−33	−81	39
Fusiform gyrus	L	454	3.218	−33	−69	−12
Fusiform gyrus	R	346	2.256	42	−63	−15
Cerebellum (VI)	R	346	3.053	27	−78	−15
Cerebellum (VII)	R	15	−2.236	12	−72	−9
Precentral gyrus	L	34	2.454	−48	−3	36
Precentral gyrus	R	21	2.033	48	3	36
Posterior-medial frontal	L	11	2.266	−9	12	63
Posterior-medial frontal	R	18	2.112	9	3	63
Superior-medial gyrus	R	15	2.252	15	18	60

It can be seen from Figure 3 and Table 2 that the positive activation areas of brain functional regions with circular shapes mainly involve the suboccipital lobe, fusiform gyrus, suboccipital gyrus and cerebellar VI, followed by the anterior central gyrus, posterior medial frontal lobe and superior frontal gyrus; The negative activation areas mainly involve the middle occipital gyrus and cerebellar VI. Both the left and right brains have positive and negative activation reactions, but the primary activation reaction is positive. As shown in Table 2, the voxel group information activated by circular shapes includes 454 voxels when the activation range is the largest, which are concentrated in the suboccipital lobe and fusiform gyrus of the left brain, followed by 346 voxels, which are concentrated in the suboccipital lobe, cerebellar VI and fusiform gyrus of the right brain. The negative activation was mainly in the middle occipital gyrus of the left brain, containing 17 voxels; Cerebellum VI of the right brain contains 15 voxels.

**FIGURE 4:**
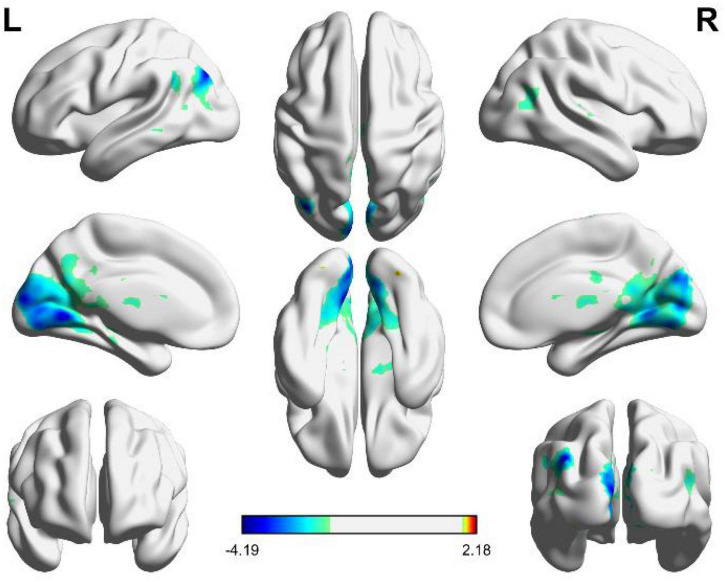
Brain functional areas activated by angular shapes. The color bar indicates the size of the activation intensity. The values indicate positive or negative activation.

As shown in Figure 4, the independent brain regions are activated by angular patterns, and the details of activated voxel groups are shown in Table 3.

**TABLE 3:** Clusters of brain areas activated by angular shapes.

Labels	Brain side	Voxels	*t*	MNI		
				***x***	***y***	***z***
Middle occipital gyrus	L	9551	−4.190	−39	−78	36
Fusiform gyrus	L	44	2.184	−30	−87	−12
Cerebellum (VI)	R	28	2.177	27	−81	−18
Cerebellum (crus 1)	L	13	−2.184	−42	−60	−27
Calcarine gyrus	R	9551	−2.027	12	−75	21
Cuneus	L	9551	−4.171	3	−75	30
Lingual gyrus	L	9551	−3.993	−9	−63	0
Superior medial gyrus	L	31	−3.244	3	36	54
Angular gyrus	L	16	−2.499	−42	−63	51
Angular gyrus	R	14	−2.043	57	−57	36
Middle frontal gyrus	L	17	−2.238	−30	36	42
Superior frontal gyrus	L	13	−1.953	−21	51	30
Middle temporal gyrus	R	24	−1.993	60	−6	−18

It can be seen from Figure 4 and Table 3 that the positive activation of functional brain areas by angular shapes mainly involve the fusiform gyrus and cerebellum, the negative activation mainly involves the middle occipital gyrus, cuneus and lingual gyrus, followed by the superior medial gyrus, middle frontal gyrus, middle temporal gyrus and angular gyrus. The left and right brains have positive and negative activation, mainly negative activation. As shown in Table 3, voxel group information is activated by angular shapes. The largest range is concentrated in the middle occipital gyrus, cuneus and lingual gyrus, including 9551 voxels, which are all negatively activated. Positive activation is mainly in the fusiform gyrus of the left brain, containing 44 voxels, and the cerebellar region of the right brain, containing 28 voxels.

To sum up, the brain activation of circular shapes is mainly positive, while the activation of angular shapes is mainly negative. Viewing circular shapes and angular shapes may involve different neural computations.

## Discussion

Circular shapes tend to be perceived as positive, while angular shapes tend to be perceived as negative (Aronoff et al., 1988, 1992; Bar and Neta, 2016). Previous studies have indicated the non-arbitrary correspondence between circular and angular shapes and various attributes (Spence et al., 2010; Spence, 2011; Hanson-Vaux et al., 2013; Fryer et al., 2014). However, there are few studies on the specific brain regions involving different geometric shapes in brain processing. The brain regions involved in the processing of circular shapes and angular shapes and their differences are unclear. Thus, this study used a simple shape-viewing task to study the activation responses of brain regions when viewing these two different types of shapes through fMRI.

The results showed that the brain regions activated by geometric shapes were mainly in the cuneus and cerebellar VI region. Cuneus is involved in basic visual processing, and Cerebellar VI is related to motor planning and coordination (Guell et al., 2018). The cerebellum involvement might be explained by the reasons for motor affordances of shapes (Lewis et al., 2003). It should be noted that the different stages of visual processing involve the activity coming from the pre-cuneus, lateral occipital and posterior inferotemporal cortex (Coppen et al., 2018). Moreover, the activation of brain regions involved the fusiform, suboccipital, and middle occipital gyrus. This is because the function of the fusiform gyrus is related to object and face recognition (Keshav and Hof, 2013). The middle occipital gyrus is the brain region of early visual processing, mainly responsible for extracting shapes and external visual features of words and objects (Beharelle and Small, 2016).

For the brain activation of circular and angular shapes, circular shapes are mainly positive, while angular shapes are mainly negative. These observations suggest that the brain’s basis of circular and angular shapes processing involves different computations. The different, often neighboring, brain regions are more active for round versus for sharp surface, which also supports the different computation theroy, or a gradient-of-representation theories (Freiwald, 2020; Hesse and Tsao, 2020). However, no significant brain area was detected comparing circular and angular shapes. This result was different from our expectations. One reason could be that the experiment design was not sensitive enough. In future, we should record the rating of the different shapes during fMRI scanning so that the valence ratings of the circular and angular shapes should be confirmed to be different.

We also hypothesized that geometry is processed in the primary visual cortex and brain areas such as higher emotion. The current study emphasizes the role of emotion processing in angular and curved correspondence, which is also supported by neuroscience evidence, which emphasizes the role of emotion in multisensory perception and shape perception (Drevets et al., 1998; Vuilleumier, 2005; Bar and Neta, 2007; Palmer et al., 2013). However, the current study did not find amygdala activation in the angular-shape or circular-shape conditions. One explanation is that simple geometric shapes are more abstract and have weaker emotional cues than facial expressions or scenes with emotional meanings. Therefore, in future studies, we can consider a more active task and choosing more specific or more emotional geometric figures, which may lead to more different results.

One limitation of this study is that it adopts a simple shape-viewing task. Moreover, in the geometric sense, only two types of figures are simple and have the same topological properties. Other than the curvature of the shape, the spatial frequencies, inked size and local contrast should be considered in future experiment design. In further studies, we may consider adopting a more sensitive experimental paradigm to identify the brain activation of circular and angular shapes, and localize the ROI at amygdala and cuneus. Moreover, the number of subjects in this study is relatively small. More samples in the future study will guarantee better reliability and validity.

## Data availability statement

The original contributions presented in this study are included in the article/Supplementary material, further inquiries can be directed to the corresponding authors.

## Ethics statement

The studies involving human participants were reviewed and approved by the Ethics Committee of the No. 2 People’s Hospital of Changshu. The patients/participants provided their written informed consent to participate in this study.

## Author contributions

LW, XL, LiH, QD, and PL conceived and designed the experiments and contributed to the writing of the manuscript. YL, LuH, and WS performed the experiments and analyzed the data. All authors contributed to the article and approved the submitted version.

## Footnotes

1
http://www.fil.ion.ucl.ac.uk/spm

2
http://rfmri.org/dpabi

3
http://www.mathworks.com
