# Supplementary material for: An fMRI study of visual geometric shapes processing
Source: Front Neurosci. 2023 Mar 16;17:1087488. doi: 10.3389/fnins.2023.1087488 (PMC10062448; doi:10.3389/fnins.2023.1087488)
Supplement: Supplementary file 1 [file Data_Sheet_1.PDF]

Supplemental Materials

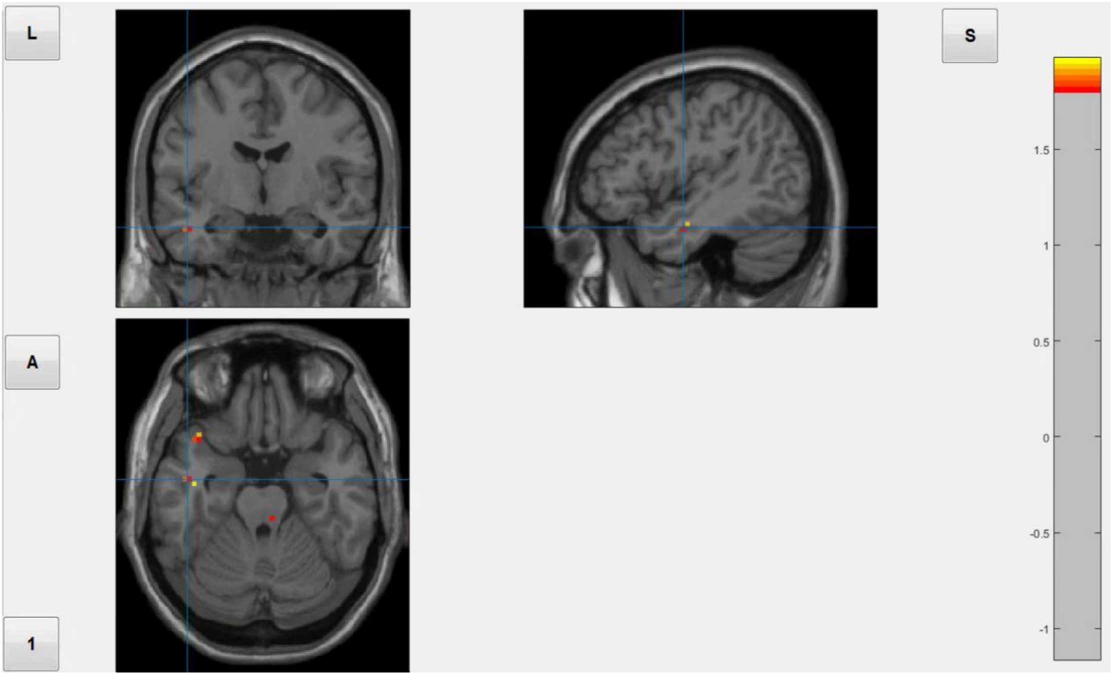

Figure S1 Brain activation regions of circular shapes vs. angular shapes. The colour bar indicates the size of the activation intensity. The values indicate positive or negative activation.

Table S1 Clusters of brain activation areas for circular shapes vs. angular shapes

Labels	Voxels	*t*	MNI		
			x	*y*	*z*
Inferior Temporal Gyrus L	6	1.977	-42	-9	-24
Temporal Pole L	3	1.944	-39	21	-24
Cerebelum (Crus 1) L	1	1.825	-9	-93	-15

Note: L: left brain, R: right brain. *t* value shows the mean difference of activation.
